# Evolutionarily distinct strategies for the acquisition of inorganic carbon from seawater in marine diatoms

**DOI:** 10.1093/jxb/erx102

**Published:** 2017-04-08

**Authors:** Yoshinori Tsuji, Anggara Mahardika, Yusuke Matsuda

**Affiliations:** Department of Bioscience, School of Science and Technology, Kwansei Gakuin University, Gakuen, Sanda, Hyogo, Japan

**Keywords:** Bicarbonate transporter, CO_2_ assimilation, CO_2_-concentrating mechanism, external carbonic anhydrase, marine phytoplankton, photosynthesis

## Abstract

The acquisition of dissolved inorganic carbon (DIC) in CO_2_-limited seawater is a central issue to understand in marine primary production. We previously demonstrated the occurrence of direct HCO_3_^–^ uptake by solute carrier (SLC) 4 transporters in a diatom, a major marine primary producer. Homologs of SLC are found in both centric and pennate marine diatoms, suggesting that SLC transporters are generally conserved. Here, the generality of SLC-mediated DIC uptake in diatoms was examined using an SLC inhibitor, diisothiocyano-2,2ʹ-stilbenedisulfonic acid (DIDS), and an inhibitor of external carbonic anhydrase, acetazolamide. DIDS suppressed high-DIC-affinity photosynthesis in the pennate diatom *Phaeodactylum tricornutum* and the centric diatom *Chaetoceros muelleri*, but there was no effect on either the pennate *Cylindrotheca fusiformis* or the centric *Thalassiosira pseudonana*. Interestingly, the DIC affinity of DIDS-insensitive strains was sensitive to treatment with up to 100 μM acetazolamide, displaying a 2–4-fold increase in *K*_0.5_[DIC]. In contrast, acetazolamide did not affect the DIDS-sensitive group. These results indicate the occurrence of two distinct strategies for DIC uptake—one primarily facilitated by SLC and the other being passive CO_2_ entry facilitated by external carbonic anhydrase. The phylogenetic independence of these strategies suggests that environmental demands drove the evolution of distinct DIC uptake mechanisms in diatoms.

## Teaser

The uptake of dissolved inorganic carbon by marine diatoms is facilitated by solute carrier transporters or external carbonic anhydrases. The occurrence of these distinct strategies is unrelated to phylogeny.

## Introduction

Marine diatoms are major primary producers in the ocean and they are estimated to contribute up to 20% of annual global carbon fixation (Falkowski *et al.*, 1998; Smetacek, 1999). Thus, diatom primary production is a major driving force for incorporating organic carbon into the ocean ecosystem and is central to the biological carbon pump from the surface to deep sea (Falkowski *et al.*, 1998; Smetacek, 1999). Despite the ecological importance of marine diatoms, the molecular basis underlying active carbon fixation by marine diatoms is not fully understood. In seawater, the dominant form of dissolved inorganic carbon (DIC) is HCO_3_^–^ (2 mM), while the concentration of CO_2_ is low (up to 15 μM under the present atmosphere at >15 °C) (Raven, 1994). The half-saturation constant (*K*_m_) values for CO_2_ of ribulose-1,5-bisphosphate carboxylase/oxygenase (Rubisco) in marine diatoms range from 23 to 65 μM at 25 °C, much higher than the CO_2_ concentration ([CO_2_]) in seawater (Badger *et al.*, 1998; Young *et al.*, 2016). In addition, owing to the slow rates of dissolved CO_2_ diffusion and the spontaneous equilibrium between CO_2_ and HCO_3_^–^, CO_2_-dependent DIC acquisition can become a rate-limiting step of photosynthesis. To avoid such problems, most marine phytoplankton, including diatoms, developed the CO_2_-concentrating mechanism (CCM) by which [CO_2_] in the vicinity of Rubisco is elevated by active accumulation of DIC from the surrounding medium, resulting in the establishment of high-DIC-affinity photosynthesis (Badger *et al.*, 1998; Giordano *et al.*, 2005; Kaplan and Reinhold, 1999; Matsuda *et al.*, 2011). A general model of CCM in eukaryotic algae comprises active DIC transport across plasma membranes and chloroplast membranes as well as carbonic anhydrase (CA)-mediated supply of CO_2_ in the vicinity of Rubisco (Giordano *et al.*, 2005).

Previous physiological studies have demonstrated occurrences of both active CO_2_ and HCO_3_^–^ uptake in marine diatoms (Burkhardt *et al.*, 2001; Matsuda *et al.*, 2001; Nimer *et al.*, 1997; Rost *et al.*, 2003). However, the molecular identity of the HCO_3_^–^ transporter was unknown until Nakajima *et al.* (2013) demonstrated that the marine pennate diatom *Phaeodactylum tricornutum* uses solute carrier (SLC) 4 transporters for direct uptake of HCO_3_^–^ from seawater across the plasma membrane. Transcription of three SLC4 genes in *P. tricornutum* was highly CO_2_ responsive and the nature of one of these, SLC4-2, was characterized by generating overexpressing strains. When SLC4-2 was constitutively overexpressed in *P. tricornutum* under high CO_2_ conditions in which the endogenous CCM was suppressed (Harada *et al.*, 2006; Hennon *et al.*, 2015; Matsuda *et al.*, 2011; Nakajima *et al.*, 2013; Ohno *et al.*, 2012; Tanaka *et al.*, 2016), cells showed increased affinity for DIC and highly halophilic Na^+^-dependent HCO_3_^–^ uptake (Nakajima *et al.*, 2013). To maintain the optimum uptake rate, PtSLC4-2 requires more than 100 mM Na^+^ (Nakajima *et al.*, 2013), meaning that it is one of the most halophilic Na^+^-dependent HCO_3_^–^ transporters so far identified in microalgae and cyanobacteria (Price *et al.*, 2004; Shibata *et al.*, 2002). Such a high Na^+^ requirement suggests specific adaptation of the diatom CCM to a high-salinity environment.

Apart from the diatom SLC4 system, in a freshwater green alga, *Chlamydomonas reinhardtii*, transporters other than SLC4, such as high light activated 3 (HLA3) and low CO_2_-inducible protein 1 (LCI1), are known to facilitate HCO_3_^–^ uptake across the plasma membrane (Duanmu *et al.*, 2009; Ohnishi *et al.*, 2010; Yamano *et al.*, 2015). The occurrence of independent transporters in different lineages strongly suggests convergent evolution of DIC uptake systems among eukaryotic algae.

The possibility of convergent evolution of DIC uptake has been suggested even within the diatom clade. Although functional characterization of SLC4 has been performed only in *P. tricornutum*, genomic information indicates the possibility of SLC4-mediated HCO_3_^–^ transport in *Thalassiosira pseudonana* (Nakajima *et al.*, 2013), suggesting a common mechanism of HCO_3_^–^ transport mediated by SLC4s. On the other hand, substantial differences in the subtypes and subcellular localization of CAs in *P. tricornutum* (Tachibana *et al.*, 2011) and *T. pseudonana* (Samukawa *et al.*, 2014) indicate that the CCM in these diatom species may be distinct, suggesting different evolutionary trajectories.

Diatoms have multiple CAs, which are classified into different subtypes based on amino acid sequence and localized in different subcellular compartments. These CAs are hypothesized to facilitate the efficient mobilization of DIC to Rubisco and prevent leakage of CO_2_ (Harada *et al.*, 2005; Kikutani *et al.*, 2012; Samukawa *et al.*, 2014; Tachibana *et al.*, 2011; Tanaka *et al.*, 2005). In *P. tricornutum*, two pyrenoidal β-CAs are considered to be key components of the CCM; *T. pseudonana* has neither β-CA nor pyrenoid-localized CA (Samukawa *et al.*, 2014; Tachibana *et al.*, 2011). Another divergence between the species is the presence of a cytosol-localized γ-CA in *T. pseudonana*; there is no evidence for a cytosolic CA in *P. tricornutum*. Additionally, *T. pseudonana* has many δ-CAs and a ζ-CA, while these subtypes are absent in *P. tricornutum*. In *T. pseudonana*, one δ-CA and one ζ-CA are localized in the periplasmic space and their transcription is activated under low-CO_2_ conditions, suggesting the involvement of these external CAs (eCAs) in DIC acquisition. There is currently no molecular evidence for eCAs in *P. tricornutum* (Samukawa *et al.*, 2014; Tachibana *et al.*, 2011), lending further evidence for the existence of fundamentally different DIC uptake strategies in these diatoms. Recently, a novel CA, named θ-CA, located in the lumen of the pyrenoid-penetrating thylakoid of *P. tricornutum* has been identified and revealed to have an essential role in efficient CCM and growth (Kikutani *et al.*, 2016), but the localization and role of θ-CA in *T. pseudonana* has yet to be examined.

It has been suggested that eCAs facilitate DIC uptake across the plasma membrane in many microalgae (Aizawa and Miyachi, 1986). When cytosolic [CO_2_] is lower than that in the external media, CO_2_ enters the cytosol by diffusion. eCA helps to maintain diffusive CO_2_ entry across the plasma membrane by facilitating a constant supply of CO_2_ from HCO_3_^–^ at the periplasmic space (Aizawa and Miyachi 1986; Hopkinson *et al.*, 2011; Matsuda *et al.*, 2017). While alternative roles of eCA, such as pH regulation, have been proposed, eCA activity primarily responded to the CO_2_ concentration in medium, rather than pH, in the marine-centric diatoms *T. pseudonana* and *Thalassiosira weissflogii* (Hopkinson *et al.*, 2013), supporting the idea that eCA enhances CO_2_ supply in these marine diatoms. eCA activity has been detected in many marine diatoms other than these two species (Burkhardt *et al.*, 2001; Hopkinson *et al.*, 2013; Martin and Tortell, 2008; Nimer *et al.*, 1997; Samukawa *et al.*, 2014), suggesting that eCA-mediated CO_2_ uptake is an alternative way to increase DIC availability besides direct HCO_3_^–^ uptake.

While the molecular basis of CCM in marine diatoms has been studied mainly in *P. tricornutum*, careful examination is needed to generalize the model of CCM to other marine diatoms. Diatoms comprise more than 10^5^ species (Mann and Droop, 1996) and there is extensive diversity in their morphological and physiological properties (Admiraal *et al.*, 1986; Kooistra *et al.*, 2007). This diversity is one of the reasons explaining their wide distribution in the world’s oceans, from polar to tropical regions; such diversity in living environment may also be reflected in the diversity of the CCM. As mentioned earlier, significant differences in the subtypes and locations of CAs between *P. tricornutum* and *T. pseudonana* suggest that they employ different CCM strategies, which may have differentiated during the diversification of the pennate and centric groups or may have arisen as a result of convergent evolution, utilizing a set of components obtained from partially (or totally) independent origins.

In the present study, a comparative physiological analysis was carried out to investigate the spectrum of CCM strategies among four marine diatom strains—two raphid pennate diatoms (*P. tricornutum* and *Cylindrotheca fusiformis*) and two multipolar centric diatoms (*T. pseudonana* and *Chaetoceros muelleri*). By examining the effect of specific SLC4 and eCA inhibitors on photosynthetic affinity for DIC and maximum photosynthetic rate, we demonstrate the occurrence of two distinct strategies for the initial uptake of DIC from the surrounding seawater.

## Materials and methods

### Strains and culture conditions

Organisms used in this study were *P. tricornutum* UTEX642, *C. fusiformis* NEPCC417, *T. pseudonana* CCMP1335, and *C. muelleri* CCMP1316. All diatoms were grown in F/2 artificial seawater (F/2ASW) (Guillard and Ryther, 1962; Harrison *et al.*, 1980) supplemented with 10 nM sodium selenite. Diatoms except *T. pseudonana* were grown under continuous illumination at 80 μmol photons m^–2^ s^–2^ and *T. pseudonana* was grown at 20 μmol photons m^–2^ s^–2^. All cells were grown under constant bubbling with atmospheric air at 20 °C. Growth was monitored by measuring optical density at 730 nm (OD_730nm_) and cells at logarithmic growth phase (OD_730nm_ = 0.2–0.4) were used for all experiments. Chlorophyll concentration was determined according to Jeffrey and Humphrey (1975).

### Measurement of photosynthetic parameters

Photosynthetic DIC affinity and maximum rate were determined by using a Clark-type oxygen electrode (Hansatech Instruments Ltd, Norfolk, UK) according to Nakajima *et al.* (2013). For measurement, all diatoms were suspended in DIC-free F/2ASW (pH8.2, buffered with 10 mM Tris-HCl) at 10 μg Chl *a* ml^–1^. Cells were maintained in a sealed water-jacketed acrylic chamber and illuminated to allow cells to reach the CO_2_ compensation point (CP). Then, the DIC concentration ([DIC]) at the CP was determined by gas chromatography (GC-8A, Shimadzu Co., Kyoto, Japan) equipped with a methanizer and flame ionization detector (GC-FID) (Birmingham and Colman 1979). The O_2_ evolution rate at various [DIC] was measured by injecting a known amount of NaHCO_3_ as a substrate. Maximum photosynthetic O_2_ evolution rate (*P*_max_) and half-saturation constant for DIC (*K*_0.5_[DIC]) were determined by rectangular-hyperbola fitting to data with non-linear least squares regression. Apparent photosynthetic conductance (APC), which is a coefficient representing changes in photosynthetic rate as the [DIC] tends toward zero, was calculated from the initial slope of the O_2_ evolution rate versus [DIC] curve (Johnston *et al.*, 1992). Light intensity used for measurements was as follows: 300 μmol photons m^–2^ s^–2^ for *P. tricornutum*, *C. fusiformis*, and *C. muelleri*, and 200 μmol photons m^–2^ s^–1^ for *T. pseudonana*. Light intensity was set at about saturation point and no inhibitory effects were observed throughout the experiment (data not shown). 4,4ʹ-Diisothiocyano-2,2ʹ-stilbenedisulfonic acid (DIDS), an inhibitor of SLC4, and acetazolamide (AZA), a weakly permeable inhibitor of CA (Moroney *et al.*, 1985), were dissolved in DMSO and ethanol, respectively. As mock treatment, 1% (v/v, final concentration) DMSO or 1% (v/v, final concentration) ethanol was added.

### Determination of DIC uptake rate

DIC uptake and efflux of CO_2_ were measured according to Nakajima *et al.* (2013) with modification. Cells in logarithmic growth phase were harvested and resuspended in DIC-free F/2ASW at a concentration of 20 μg Chl *a* ml^–1^. Cells were then transferred to a 1.5 ml microtube equipped with a filter cartridge (Nanosep MF GHP, pore size 0.45 μm, Pall Corp., NY, USA) and pre-incubated under illumination for 30 s. The reaction was initiated by addition of NaHCO_3_ at a final concentration of 100 μM (for *P. tricornutum* and *C. muelleri*) or 200 μM (for *C. fusiformis* and *T. pseudonana*). After the various incubation periods, the filter cartridge containing the cell suspension was centrifuged to allow separation of medium and cells. Then, the remaining DIC in the medium at each time point was immediately measured by GC-FID. The initial depletion rate of DIC during the first 240 s of the light period was calculated as the net DIC uptake rate (*N*_DIC_). The efflux rate of DIC (*E*_C_) was calculated from the CO_2_ evolution rate in the dark immediately after 240 s of light exposure. Gross DIC uptake rate (*G*_DIC_) was calculated from *N*_DIC_ and *E*_C_ using the DIC flux model presented by Nakajima *et al.* (2013).

To examine the energy source of DIC uptake, we used 3-(3,4-dichlorophenyl)-1,1-dimethylurea (DCMU) as an inhibitor of linear electron transport (LET). Additionally, carbonyl cyanide-*m*-chlorophenyl hydrazone (CCCP) was used to inhibit proton gradient and membrane potential-coupled ATP synthesis. These inhibitors were dissolved in DMSO and then added to the cell suspension immediately before the pre-incubation period. The final concentration of DCMU and CCCP was 2 μM and 10 μM, respectively. In control experiments, 1% (v/v) DMSO was added as a mock treatment. In *C. fusiformis* and *T. pseudonana*, initial [DIC] was set higher (200 μM) than for the other two diatoms (100 μM), since *C. fusiformis* and *T. pseudonana* have lower affinity for DIC and showed only weak DIC uptake activity at 100 μM DIC.

## Results

### Effects of inhibitors of SLC and extracellular CA on pennate diatoms

**Fig. 1.**
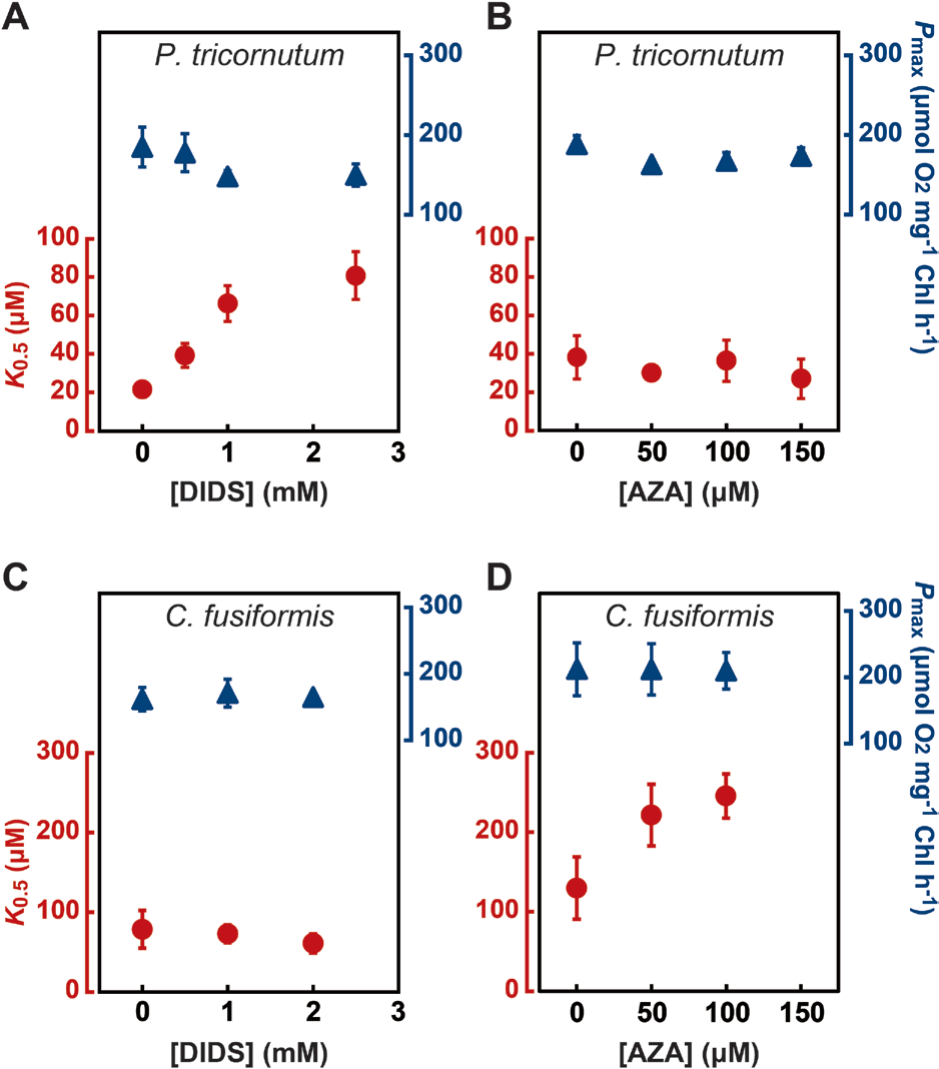
Effects of DIDS and AZA on pennate diatoms. Photosynthetic parameters (*K*_0.5_[DIC] and *P*_max_) in air-grown *P. tricornutum* (A, B) and *C. fusiformis* (C, D) were determined in the presence of various concentrations of DIDS (A, C) or AZA (B, D). Circles, *K*_0.5_; triangles, *P*_max_. Values represent means ± SD of three or four biological replicates. Panel A was generated from data in Nakajima *et al.* (2013).

To examine the contribution of SLC4 transporters to DIC acquisition in pennate and centric diatoms, we tested the effect on *K*_0.5_[DIC] and *P*_max_ of the commonly used SLC4 HCO_3_^–^ uptake inhibitor DIDS and the weakly permeable CA inhibitor AZA relative to the 1% (v/v) DMSO or 1% (v/v) ethanol-treated mock cells with four diatom species: two raphid pennates, *P. tricornutum* and *C. fusiformis*, and two multipolar centrics, *T. pseudonana* and *C. muelleri*. DMSO and ethanol had no effect on *K*_0.5_[DIC] and *P*_max_ (data not shown). *P. tricornutum* displayed a significant sensitivity to DIDS. The *K*_0.5_[DIC] increased with increasing concentrations of DIDS, reaching a 4-fold increase at 2.5 mM DIDS relative to the non-treated cells (Fig. 1A and Supplementary Table S1 at JXB online). Changes in APC, an index of photosynthetic DIC affinity calculated from the initial slope of the photosynthetic O_2_ evolution versus [DIC] curve (Johnston *et al.*, 1992), and [DIC] at the CO_2_ CP, are listed in Table 1. In *P. tricornutum*, changes in [DIC] at the CO_2_ CP were not significant, but APC was decreased 4-fold in the presence of 2.5 mM DIDS (Table 1). In contrast, when the role of eCA was examined by treatment with AZA, *K*_0.5_[DIC] and APC were unaffected in *P. tricornutum* (Fig. 1B, Table 1, Supplementary Tables S1 and S2), consistent with a previous report showing no eCA in this diatom (Satoh *et al.*, 2001). These results suggest that DIC uptake of *P. tricornutum* is primarily supported by DIDS-sensitive active transport of HCO_3_^–^, most probably by SLC4 as indicated by Nakajima *et al.*, 2013.

**Table 1.** Effects of inhibitors on apparent photosynthetic conductance (APC) and [DIC] at the CO_2_ compensation point (CO_2_ CP) in pennate diatoms

Species	Inhibitor	Concentration of inhibitor	APC [μmol O _ 2 _ mg ^ –1 ^ Chl h ^ –1 ^ (μM DIC) ^ –1 ^ ]	[DIC] at CO _ 2 _ CP (μM)^*a*^
*P. tricornutum*	DIDS^*b*^	0 mM	4.80 ± 0.62	9.1 ± 4.3
		0.5 mM	2.83 ± 0.93	9.4 ± 5.4
		1 mM	1.47 ± 0.31	7.6 ± 4.6
		2.5 mM	1.17 ± 0.12	11.9 ± 3.1
	AZA	0 μM	3.13 ± 0.38	nd
		50 μM	3.07 ± 0.80	nd
		100 μM	2.70 ± 0.18	nd
		150 μM	2.73 ± 0.54	nd
*C. fusiformis*	DIDS	0 mM	1.46 ± 0.54	5.3 ± 3.5
		1 mM	1.47 ± 0.16	5.4 ± 0.7
		2 mM	1.58 ± 0.26	6.4 ± 2.8
	AZA	0 μM	1.08 ± 0.51	nd
		50 μM	0.73 ± 0.2	nd
		100 μM	0.62 ± 0.13	nd

nd, not determined. Values represent means ± SD of three or four replicates.

^*a*^[DIC] in the presence of AZA could not be determined because AZA was dissolved in ethanol, which interferes with the detection of DIC by GC-FID.

^*b*^Values of DIDS experiments in *P. tricornutum* were calculated from data in Nakajima *et al.* (2013).

The response of another raphid pennate diatom, *C. fusiformis*, to DIDS and AZA treatment was opposite to that observed in *P. tricornutum*. In *C. fusiformis*, values for *K*_0.5_[DIC], APC, and [DIC] at CO_2_ CP were not affected by treatment with DIDS at concentrations up to 2 mM (Fig. 1C, Table 1, Supplementary Tables S1 and S2). In contrast to the effect of DIDS, treatment with100 μM AZA doubled the *K*_0.5_[DIC] value and decreased the APC value to 50% compared with corresponding values in non-treated cells (Fig. 1D, Table 1). These data strongly suggest that eCA-mediated DIC uptake is the major process in the DIDS-insensitive pennate *C. fusiformis*, as an alternative strategy to direct HCO_3_^–^ uptake. Neither DIDS nor AZA had a significant effect on the *P*_max_ of either diatom (Fig. 1A–D), indicating that the effect is specific to the CCM, and there was no impact of these agents on the central machinery of the photosystem or the Calvin cycle.

### Effects of inhibitors of SLC and extracellular CA on centric diatoms

**Fig. 2.**
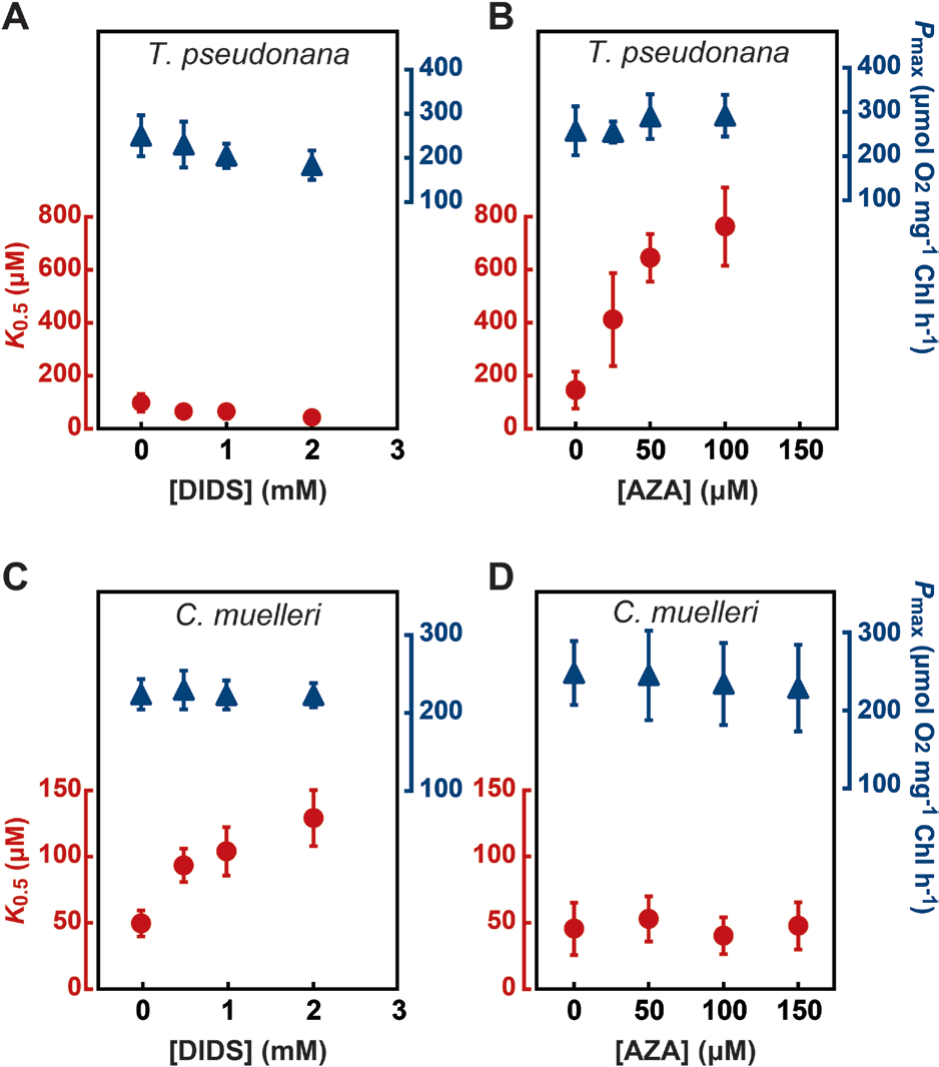
Effects of DIDS and AZA on centric diatoms. Photosynthetic parameters (*K*_0.5_[DIC] and *P*_max_) in *T. pseudonana* (A, B) and *C. muelleri* (C, D) were determined in the presence of various concentrations of DIDS (A, C) or AZA (B, D). Circles, *K*_0.5_; triangles, *P*_max_. Values represent means ± SD of three or four biological replicates.

To further explore the evolutionary relationship between DIC uptake strategies, the same treatment and analysis were carried out on two multipolar centric marine diatoms, *T. pseudonana* and *C. muelleri*. For *T. pseudonana*, DIDS treatment at concentrations up to 2 mM did not alter *K*_0.5_[DIC] (Fig. 2A, Supplementary Tables S1 and S2); the parameters APC and [DIC] at CO_2_ CP were similarly not affected by DIDS (Table 2). Similar to the results in *C. fusiformis* (Fig. 1C), this suggested that HCO_3_^–^ uptake by SLC4 is not the dominant DIC uptake strategy in *T. pseudonana*. In sharp contrast, AZA treatment with concentrations up to 100 μM increased *K*_0.5_[DIC] more than 5-fold relative to the value in non-treated cells in *T. pseudonana* (Fig. 2B, Supplementary Tables S1 and S2), again resembling the DIC characteristics in *C. fusiformis* (Fig. 1D). At concentrations lower than 100 μM AZA, APC also decreased to 25% relative to the non-treated cells in *T. pseudonana* (Table 2), clearly indicating that the initial DIC uptake strategy in *T. pseudonana* is highly dependent on eCA and the subsequent permeation of CO_2_ across the plasma membrane.

In a second centric diatom species, *C. muelleri*, the responses to the two inhibitors were the opposite of those observed in *T. pseudonana*, and resembled the results seen in the pennate species *P. tricornutum* (Fig. 1A, B). *K*_0.5_[DIC] increased 2.6-fold after treatment with 2 mM DIDS (Fig. 2C, Supplementary Table S1) but AZA did not affect *K*_0.5_[DIC] in *C. muelleri* at concentrations up to 150 μM (Fig. 2D, Supplementary Table S1). APC decreased 2.5-fold and [DIC] at CO_2_ CP doubled in the presence of 2 mM DIDS relative to the corresponding values in non-treated cells (Table 2), strongly suggesting that *C. muelleri* utilizes a direct HCO_3_^–^ uptake strategy for the initial acquisition of environmental DIC, similar to *P. tricornutum*. In both centric diatoms, *P*_max_ was fairly stable under all concentrations of DIDS and AZA, indicating no impact of either agent on the central photosynthesis machinery. In addition, the inhibitory effects of DIDS and AZA on *K*_0.5_[DIC] were concentration-dependent and near-saturated at the highest concentration used (2.5 mM for DIDS and 100 μM for AZA). These concentrations of DIDS and AZA have been commonly used in previous studies (Huertas *et al.*, 2000; Moroney *et al.*, 1985; Nakajima *et al.*, 2013; Satoh *et al.*, 2001). Furthermore, Nakajima *et al.* (2013) showed that *P. tricornutum* engineered for high DIC uptake conferred by constitutive expression of PtSLC4-2 was efficiently inhibited by DIDS at the concentrations used here, indicating that this agent specifically targets SLCs. Given these considerations, the decreased *K*_0.5_[DIC] values in response to DIDS and AZA treatments (Fig. 1 and Fig. 2) were considered to be the result of specific inhibition of SLC4 and eCA rather than ‘off-target’ side effects.

**Table 2.** Effects of inhibitors on apparent photosynthetic conductance (APC) and [DIC] at the CO_2_ compensation point (CO_2_ CP) in centric diatoms

Species	Inhibitor	Concentration of inhibitor	APC [μmol O _ 2 _ mg ^ –1 ^ Chl h ^ –1 ^ (μM DIC) ^ –1 ^ ]	[DIC] at CO _ 2 _ CP (μM)^*a*^
*T. pseudonana*	DIDS	0 mM	1.41 ± 0.38	3.7 ± 0.8
		0.5 mM	1.84 ± 0.53	4.2 ± 0.9
		1 mM	1.57 ± 0.29	4.4 ± 1.8
		2 mM	1.91 ± 0.13	3.9 ± 2.0
	AZA	0 μM	1.11 ± 0.27	nd
		25 μM	0.40 ± 0.24	nd
		50 μM	0.29 ± 0.04	nd
		100 μM	0.26 ± 0.12	nd
*C. muelleri*	DIDS	0 mM	2.36 ± 0.34	1.6 ± 0.8
		0.5 mM	1.53 ± 0.28	1.7 ± 0.5
		1 mM	1.14 ± 0.28	1.8 ± 0.2
		2 mM	1.02 ± 0.16	3.2 ± 0.8
	AZA	0 μM	3.33 ± 1.04	nd
		50 μM	3.15 ± 0.84	nd
		100 μM	3.60 ± 0.91	nd
		150 μM	3.31 ± 0.67	nd

nd, not determined. Values represent means ± SD of three or four replicates.

^*a*^[DIC] in the presence of AZA could not be determined because AZA was dissolved in ethanol, which interferes with the detection of DIC by GC-FID.

### Investigation of the energy source for DIC uptake

To study the energetics of DIC uptake, we tested the effects of DCMU and CCCP on DIC uptake. DCMU inhibits LET from the Quinone A socket in photosystem II to plastoquinone (PQ), suppressing NADPH biosynthesis but allowing ATP synthesis via cyclic electron flow (CEF). CCCP is an uncoupler of ATP synthase from the proton motive force, inhibiting ATP synthesis in both the mitochondria and chloroplast while not affecting electron transport chains.

**Fig. 3.**
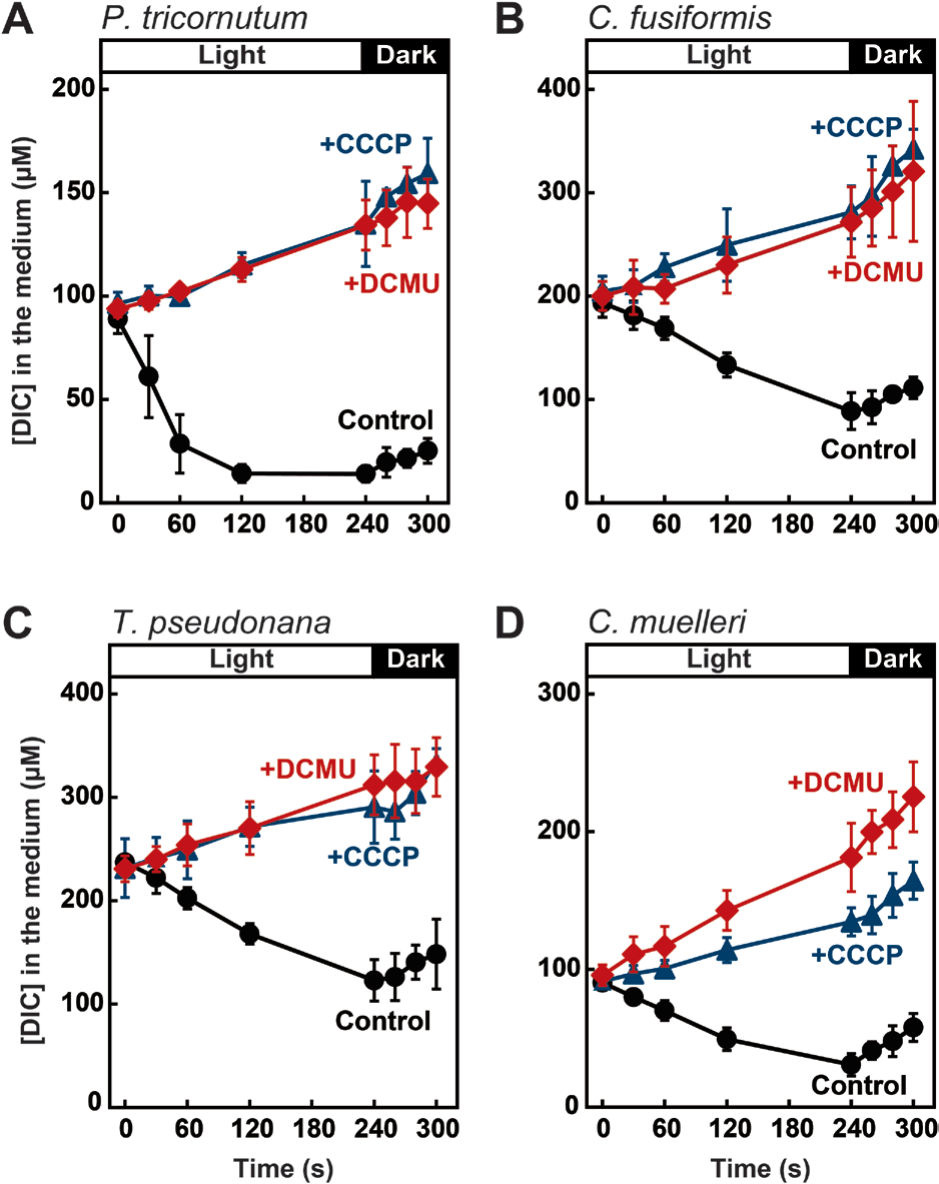
Effects of DCMU and CCCP on DIC uptake by the diatoms *P. tricornutum* (A), *C. fusiformis* (B), *T. pseudonana* (C), and *C. muelleri* (D). [DIC] in the medium was determined during 240 s of photosynthesis under light and the subsequent 60 s under dark conditions. Cells were pre-incubated with inhibitors for 30 s under light conditions and then photosynthesis was started by the addition of NaHCO_3_ at 0 s. Circles, control experiments; triangles, +CCCP (10 μM, final concentration); diamonds, +DCMU (2 μM, final concentration). Values represent means ± SD of three biological replicates.

In many phytoplankton, including diatoms, cells actively take up DIC and simultaneously release CO_2_ during photosynthesis (Hopkinson *et al.*, 2011; Huertas *et al.*, 2000; Nakajima *et al.*, 2013; Tchernov *et al.*, 1997, 2001). Given the occurrence of this massive carbon cycling across the plasma membrane, we measured the rate of net DIC uptake (*N*_DIC_) and CO_2_ efflux (*E*_C_) to calculate the gross DIC uptake rate (*G*_DIC_) according to the model presented by Nakajima *et al.* (2013). In this experiment, we measured net uptake of DIC during 240 s of photosynthesis at an initial [DIC] of 100 or 200 μM under light, and then measured CO_2_ efflux immediately after turning off the light. As shown in Fig. 3, net uptake of DIC was completely stopped by treatment with 2 μM DCMU or 10 μM CCCP. Values for *N*_DIC_, *E*_C_, and *G*_DIC_ are summarized in Table 3. In all four diatoms, *N*_DIC_, calculated from the initial slope of DIC depletion, was completely inhibited by treatment with DCMU and CCCP (Table 3, Fig. 3). Given the low CEF activity in diatoms (Bailleul *et al.*, 2015), most ATP would be produced by LET during photosynthesis. Inhibition of DIC uptake by CCCP treatment indicated that ATP generated by photophosphorylation might be the major energy source for DIC uptake. On the other hand, *E*_C_ was insensitive to these inhibitors, suggesting that efflux of CO_2_ from cells occurs independent of photosynthesis. As a result, *G*_DIC_, calculated as the sum of *N*_DIC_ and *E*_C_, was decreased by DCMU and CCCP treatment in all four diatoms.

**Table 3.** Effect of CCCP and DCMU on DIC uptake and efflux in four marine diatoms

Species	Parameter	Rate of uptake or efflux (μmol mg Chl^–1^ min^-1^)		
		Control	+CCCP	+DCMU
*P. tricornutum*	*N*_DIC_	6.99 ± 2.38	–0.54 ± 0.30	–0.53 ± 0.15
	*E*_C_	0.65 ± 0.61	0.79 ± 0.91	0.75 ± 0.03
	*G*_DIC_	7.64 ± 1.77	0.24 ± 1.19	0.22 ± 0.17
*C. fusiformis*	*N*_DIC_	1.16 ± 0.06	–0.67 ± 0.14	–0.60 ± 0.22
	*E*_C_	0.77 ± 0.33	2.16 ± 0.26	1.49 ± 0.71
	*G*_DIC_	1.93 ± 0.39	1.49 ± 0.40	0.94 ± 0.70
*T. pseudonana*	*N*_DIC_	2.18 ± 0.37	–0.72 ± 0.20	–1.03 ± 0.28
	*E*_C_	2.80 ± 0.88	2.17 ± 0.11	0.82 ± 0.94
	*G*_DIC_	4.97 ± 1.21	1.46 ± 0.22	–0.21 ± 1.01
*C. muelleri*	*N*_DIC_	1.70 ± 0.64	–0.57 ± 0.10	–1.07 ± 0.21
	*E*_C_	1.48 ± 0.20	1.63 ± 0.09	2.15 ± 0.21
	*G*_DIC_	3.18 ± 0.47	1.06 ± 0.14	1.08 ± 0.22

CCCP was added at a final concentration of 10 μM and DCMU at a final concentration of 2μM. *N*_DIC_, net DIC uptake rate; *E*_C_, CO_2_ efflux rate, *G*_DIC_, gross DIC uptake rate. Values represent means ± SD of three replicates.

## Discussion

### Phylogeny-independent strategies of DIC uptake in marine diatoms

In the oceanic environment, where HCO_3_^–^ is the predominant DIC species, utilization of HCO_3_^–^ can be advantageous, since [CO_2_] is much lower than the *K*_m_ [CO_2_] of Rubisco. Many diatoms can take up HCO_3_^–^ directly from the surrounding water to overcome the limitations of low CO_2_ for photosynthesis (Matsuda *et al.*, 2011, , 2017). SLC4 has previously been shown to transport HCO_3_^–^ across the plasma membrane in the pennate diatom *P. tricornutum*. Conservation of SLC4 homologs in the distantly related centric species *T. pseudonana* suggests that HCO_3_^–^ uptake by SLC4 is a common mechanism across diverse diatoms. However, our study, using a specific inhibitor for SLC4, did not support this idea. Photosynthetic affinity for DIC in two evolutionarily distinct diatoms, *C. fusiformis* and *T. pseudonana*, was unaffected by DIDS treatment (Fig. 1C, Fig. 2A, Table S2) but decreased by AZA treatment (Fig. 1D, Fig. 2B, Table S2), suggesting that eCA-mediated indirect uptake is a major route facilitating DIC uptake. Conversely, *P. tricornutum* and *C. muelleri*, which are also evolutionarily distinct species, were sensitive to DIDS treatment but unaffected by AZA, suggesting SLC4 to be a major path for DIC uptake in these species. Interestingly, all diatoms displayed sensitivity to only DIDS or AZA, with no examples of dual sensitivity or insensitivity to both agents. These results strongly suggest that marine diatoms take at least two different, independent strategies, supported mainly by SLC4 or eCA. Additionally, there was no evidence of cooperativity in one cell. Interestingly, the occurrence of these two strategies in diatom CCM is not related to their phylogenetic position. For instance, *C. fusiformis* and *T. pseudonana* were shown to be eCA-dependent, but these strains are phylogenetically very distant pennate and centric diatoms, respectively; the same holds true for the SLC4-dependent diatoms, *P. tricornutum* and *C. muelleri*. These results support the idea that strategies for DIC uptake have been established independently as diatoms adapt to their living environment, rather than by a process of phylogenetic diversification. While there is no molecular evidence of the occurrence of eCA in *P. tricornutum*, it has been reported by physiological measurement that eCA activity occurs in some strains of *P. tricornutum*, suggesting that environmental adaptation of the CCM strategy could occur even within the same species. At present, it is difficult to draw conclusions on the exact environmental factors that directed the evolution of the SLC4- or eCA-dependent strategy. One notable feature of SLC4 is a high requirement for Na^+^ in HCO_3_^–^ transport, possibly due to the Na^+^-HCO_3_^–^ co-transport mechanism (Nakajima *et al.* 2013). On the other hand, since eCA-based DIC uptake is likely to be independent of Na^+^, eCA-dependent species may possibly be adapted to a wide range of Na^+^ concentrations, or fluctuating Na^+^ conditions, such as those of estuary areas. Therefore, Na^+^ concentration might be an environmental factor that influenced the evolution of CCM.

While this study presents the occurrence of at least two different strategies in marine diatoms, our results do not exclude the possibility that diatoms employ additional DIDS- or AZA-insensitive DIC acquisition strategies. Redundancy of DIC transporters has been documented in cyanobacteria (Kaplan and Reinhold, 1999; Ogawa and Kaplan, 2003) and green algae (Duanmu *et al.*, 2009; Ohnishi *et al.*, 2010; Wang *et al.*, 2015; Yamano *et al.*, 2015), and therefore it is possible that diatoms also have other DIC uptake mechanisms.

### Possible role of SLC4 as a chloroplast DIC transporter in DIDS-insensitive species

Our primary interest is the generality of SLC4-dependent HCO_3_^–^ uptake among marine diatoms. The results of DIDS treatment showed that direct uptake by SLC4 is not universal among marine diatoms, although SLC4 homologs are widely conserved across pennate and centric diatoms. One possible function of putative SLC4 in diatoms dependent on eCA (i.e. those insensitive to DIDS) is that SLC4 is located on chloroplast membranes to facilitate DIC transport into the stroma. DIDS is a membrane-impermeable inhibitor that reacts with a specific Lys residue of SLC exposed to the outside of cells (Romero *et al.*, 2013; Spiro and Spiro, 1985). Therefore, DIDS cannot inhibit SLC4 on intracellular membranes such as the chloroplast envelope. Diatoms have a red-algal-derived chloroplast, which is surrounded by two chloroplast envelopes and an additional outer two membranes termed the chloroplastic endoplasmic reticulum. SLC4s that play a role at the chloroplast envelope and/or chloroplastic endoplasmic reticulum should be insensitive to DIDS treatment of living cells. *T. pseudonana* has putative SLC4s containing chloroplast-targeting signal (Supplementary Table S3), but the localization of these putative SLCs in eCA-dependent diatoms has yet to be determined. Unlike diatoms, the green alga *C. reinhardtii* possesses a two-layered chloroplast, and functional cooperation between plasma-membrane-localized HLA3 and chloroplast-envelope-localized LCIA in uptake of HCO_3_^–^ has been reported (Duanmu *et al.*, 2009; Wang *et al.*, 2015; Yamano *et al.*, 2015). Such cooperation between the plasma membrane and plastid system has yet to be elucidated in any secondary symbiotic alga, but the significant complexity of the four-layered chloroplastic envelope in diatoms probably includes many regulatory points in the transport of DIC from the surrounding media to the pyrenoid compartment in the chloroplast.

### Photosynthetic ATP production drives DIC uptake in marine diatoms

To reveal the energetics of DIC uptake, we tested the effects of DCMU and CCCP. DCMU inhibits photosynthetic LET in photosystem II but does not inhibit CEF around photosystem I. CCCP does not inhibit LET but dissipates both membrane potential and proton gradients, resulting in the disruption of ATP synthesis in both the chloroplast and mitochondrion (Heytler and Prichard, 1962; Terada 1981). In our experiments, both DCMU and CCCP completely inhibited *N*_DIC_ in all diatoms examined (Fig. 3). It has recently been reported that, unlike higher plants and green algae, diatoms may modulate the ATP/NADPH ratio mainly by bypassing the photochemical electron flow through a mitochondrial respiratory pathway instead of CEF (Bailleul *et al.*, 2015). Considering the very low CEF activity in diatoms reported by Bailleul *et al.* (2015), ATP synthesis in the chloroplast is mostly dependent on LET. Therefore, it is probable that DCMU completely inhibited photophosphorylation, with little contribution of a negligible amount of ATP produced by CEF. As indicated by the inhibition of *N*_DIC_ by CCCP or under dark conditions, ATP generated by photophosphorylation is a major energy source driving active HCO_3_^–^ uptake in the DIDS-sensitive group. This feature is different from green algae and cyanobacteria, in which involvement of CEF is suggested to drive DIC uptake (Sültemeyer *et al.*, 1991; Tchernov *et al.*, 2001). Negligible activity of CEF has been demonstrated in both pennate and centric diatoms and is thus considered to be a general feature (Bailleul *et al.*, 2015). Nonetheless, a recent study showed high levels of CEF in a polar diatom, *Fragilariopsis cylindrus* (Goldman *et al.*, 2015), suggesting the possibility of involvement of CEF in the CCM of some psychrophilic diatoms and lending further support toward the hypothesis of environment-specific adaptations for DIC uptake.

While further studies are needed to reveal a coupling of ATP hydrolysis and DIC uptake, ATP is presumably utilized to maintain a Na^+^ gradient across the plasma membrane in SLC4-dependent diatoms, since SLC4 is a secondary transport system that is dependent on Na^+^ for HCO_3_^–^ uptake, most likely co-transporting HCO_3_^–^ and Na^+^. In eCA-dependent diatoms, eCA-mediated CO_2_ entry is most probably passive (Hopkinson *et al.*, 2013); thus, it is unlikely that ATP hydrolysis operates in this process. However, active DIC transport across chloroplast membranes is proposed to drive the CCM of diatoms (Hopkinson, 2014), and if this is the case, ATP should be consumed to drive an active uptake of HCO_3_^–^into the chloroplast; possibly in an SLC4-dependent manner.

## Conclusion

While conservation of SLC4 in representative pennate and centric diatoms suggested generalizability of SLC4-mediated DIC uptake in the diatom CCM, here we demonstrated SLC4-dependent and eCA-dependent strategies of the diatom CCM. These two strategies are unrelated to phylogenetic position, suggesting that each marine diatom independently developed different DIC uptake mechanisms to adapt to its living environment. While molecular studies of diatom CCM are most advanced in *P. tricornutum*, the present study indicates the necessity of detailed studies on eCA-dependent marine diatoms such as *T. pseudonana* to fully understand the evolutionary adaptation of marine diatoms to different environments.

## Supplementary data

Supplementary data are available at *JXB* online.

Table S1. Effects of DIDS and AZA on photosynthetic parameters of four diatoms.

Table S2. Qualitative summary of effects of inhibitors on photosynthetic parameters.

Table S3. Predicted localization of putative SLC4s in *T. pseudonana*.

## Supplementary Material

supplementary_tables_S1_S3

## Abbreviations:

APC: apparent photosynthetic conductance
AZA: acetazolamide
CA: carbonic anhydrase
CCM: CO_2_-concentrating mechanism
CCCP: carbonyl cyanide-*m*-chlorophenyl hydrazone
CEF: cyclic electron flow
[CO_2_]: CO_2_ concentration
CP: compensation point
DCMU: 3-(3,4-dichlorophenyl)-1,1-dimethylurea
DIC: dissolved inorganic carbon
[DIC]: DIC concentration
DIDS: diisothiocyano-2,2ʹ-stilbenedisulfonic acid
*E*_C_: rate of CO_2_ efflux
eCA: external CA
F/2ASW: F/2 artificial seawater
GC-FID: gas chromatography with flame ionization detector
*G*_DIC_: rate of gross DIC uptake
*K*_0.5_[DIC]: half-saturation constant for DIC
*K*_m_: half-saturation constant
LET: linear electron transport
*N*_DIC_: rate of net DIC uptake
*P*_max_: maximum photosynthetic O_2_ evolution rate
SLC: solute carrier.
