# Supplementary material for: Evolutionarily distinct strategies for the acquisition of inorganic carbon from seawater in marine diatoms
Source: J Exp Bot. 2017 Apr 8;68(14):3949–58. doi: 10.1093/jxb/erx102 (PMC5853789; doi:10.1093/jxb/erx102)
Supplement: supplementary_tables_S1_S3 [file erx102_suppl_supplementary_tables_s1_s3.pdf]

Supplemental data (Tables S1-S3)

Table S1 Effects of DIDS and AZA on photosynthetic parameters of four diatoms. [a]1% (v/v, final conc.) DMSO was added as a mock treatment. [b]1% (v/v, final conc.) EtOH was added as a mock treatment. Values represent means ± SD of three or four biological replicates.

**Species**	**Treatment**		**Photosynthetic parameters**	
	**inhibitor**	**concentration**	$K_{0.5}$ (μM)	P_{max} (μmol O_2 mg^{-1} Chl *a* min^{-1})
P. tricornutum	DIDS	0 mM[a]	35 ± 12	195 ± 29
		0.5 mM	69 ± 21	190 ± 28
		1 mM	80 ± 11	159 ± 10
		2.5 mM	112 ± 11	167 ± 17
	AZA	0 μM[b]	38 ± 8	188 ± 11
		50 μM	30 ± 11	163 ± 3
		100 μM	36 ± 6	167 ± 11
		150 μM	27 ± 5	174 ± 10
C. fusiformis	DIDS	0 mM[a]	93 ± 17	162 ± 17
		1 mM	73 ± 9	171 ± 19
		2 mM	64 ± 11	164 ± 3
	AZA	0 μM[b]	147 ± 33	197 ± 31
		50 μM	245 ± 24	212 ± 19
		100 μM	280 ± 16	196 ± 3
T. pseudonana	DIDS	0 mM[a]	98 ± 34	250 ± 46
		0.5 mM	65 ± 23	230 ± 52
		1 mM	65 ± 26	205 ± 28
		2 mM	43 ± 11	183 ± 33
	AZA	0 μM[b]	146 ± 70	257 ± 56
		25 μM	411 ± 175	255 ± 24
		50 μM	645 ± 90	290 ± 51
		100 μM	762 ± 148	292 ± 47
C. muelleri	DIDS	0 mM[a]	50 ± 10	224 ± 19
		0.5 mM	93 ± 13	230 ± 25
		1 mM	104 ± 18	223 ± 19
		2 mM	129 ± 21	223 ± 15
	AZA	0 μM[b]	46 ± 20	248 ± 41
		50 μM	53 ± 17	245 ± 57
		100 μM	40 ± 14	234 ± 53
		150 μM	48 ± 18	229 ± 56

Table S2 Qualitative summary of effects of inhibitors on photosynthetic parameters. ↑, increased; ↓, decreased; →, unchanged; n.d., not determined.

Group	Species	Effects of DIDS on				Effects of AZA on			
		**APC**	**CO_2 CP**	$K_{0.5}$	P_{max}	**APC**	**CO_2 CP**	$K_{0.5}$	P_{max}
Pennate	*P. tricornutum*	↓	→	↑	→	→	n.d.	→	→
	C. fusiformis	→	→	→	→	↓	n.d.	↑	→
Centric	*T. pseudonana*	→	→	→	→	↓	n.d.	↑	→
	C. muelleri	↓	↑	↑	→	→	n.d.	→	→

Table S3 Predicted localization of putative SLC4s in *T. pseudonana*. Protein ID in JGI database and accession no. in GenBank database was shown with predicted localization. For the prediction of localization, HECTAR was used (Gschloessl et al. 2008). Since the N-terminal of TpSLC4-1 is not started from Met probably due to incompleteness of databases, we cannot predict its localization.

Name	Protein ID	GenBank accession no.	Predicted localization
TpSLC4-1	23678	XP_002291948	?
TpSLC4-2	270240	XP_002286207	Chloroplast
TpSLC4-3	270361	XP_002286696	Chloroplast

Reference

Gschloessl B, Guermeur Y, Cock JM. 2008. HECTAR: A method to predict subcellular targeting in heterokonts. BMC Bioinformatics **9**, 393
